# Evaluation in health: participatory methodology and involvement of municipal managers

**DOI:** 10.1590/S1518-8787.2016050006251

**Published:** 2016-07-26

**Authors:** Cristiane Andrea Locatelli de Almeida, Oswaldo Yoshimi Tanaka

**Affiliations:** I Programa de Pós-Graduação em Saúde Pública. Faculdade de Saúde Pública. Universidade de São Paulo. São Paulo, SP, Brasil; II Departamento de Prática de Saúde Pública. Faculdade de Saúde Pública. Universidade de São Paulo. São Paulo, SP, Brasil

**Keywords:** Health Manager, Decision-making, Program Evaluation, Health Services Evaluation, Participatory Evaluation, Brazilian Unified Health System, Qualitative Research

## Abstract

**OBJECTIVE:**

To analyze scopes and limits of the use of participatory methodology of evaluation with municipal health managers and administrators.

**METHODS:**

Qualitative research with health policymakers and managers of the *Comissão Intergestores Regional* (CIR – Regional Interagency Commission) of a health region of the state of Sao Paulo in Brazil. Representatives from seven member cities participated in seven workshops facilitated by the researchers, with the aim of assessing a specific problem of the care line, which would be used as a tracer of the system integrality. The analysis of the collected empirical material was based on the hermeneutic-dialectic methodology and aimed at the evaluation of the applied participatory methodology, according to its capacity of promoting a process of assessment capable to be used as a support for municipal management.

**RESULTS:**

With the participatory approach of evaluation, we were able to promote in-depth discussions with the group, especially related to the construction of integral care and to the inclusion of the user’s perspective in decision-making, linked to the search for solution to concrete problems of managers. By joint exploration, the possibility of using data from electronic information systems was opened, as well as information coming directly from the users of the services, to enhance discussions and negotiations between partners. The participants were disbelievers of the replication potential of this type of evaluation without the direct monitoring of the academy, given the difficulty of organizing the process in everyday life, already taken by emergency and political issues.

**CONCLUSIONS:**

Evaluations of programs and services carried out within the Regional Interagency Commission, starting from the local interest and facilitating the involvement of its members by the use of participatory methodologies, can contribute to the construction of integral care. To the extent that the act of evaluating stay invested with greater significance to the local actors, its involvement with the evaluations at the federal level can also be stimulated.

## RESUMO

**OBJETIVO:**

Analisar alcances e limites do uso de metodologia participativa de avaliação junto a gestores e gerentes municipais de saúde.

**MÉTODOS:**

Pesquisa qualitativa com gestores e gerentes de saúde da Comissão Intergestores Regional de uma região de saúde do estado de São Paulo. Representantes de sete municípios membros participaram de sete oficinas facilitadas pelos pesquisadores, com o objetivo de avaliar um problema específico da linha de cuidados sob a perspectiva da integralidade. A análise do material empírico coletado baseou-se na metodologia hermenêutica-dialética e visou a avaliação da metodologia participativa aplicada, segundo sua capacidade de promover um processo de avaliação passível de ser utilizado como suporte à gestão municipal.

**RESULTADOS:**

Com a abordagem participativa de avaliação, foi possível promover debates em profundidade com o grupo, principalmente relacionados à construção da integralidade da atenção e à inclusão da perspectiva do usuário na tomada de decisão, de forma vinculada à busca de solução para problemas concretos dos gestores. Mediante a exploração conjunta, foi aberta a possibilidade de utilização de dados advindos dos sistemas eletrônicos de informação, bem como de informações advindas diretamente dos usuários dos serviços, para enriquecer debates e negociações entre parceiros. Os participantes se mostraram descrentes do potencial de replicação deste tipo de avaliação sem o acompanhamento direto da academia, dada a dificuldade de organização do processo no cotidiano, já tomado por questões emergenciais e políticas.

**CONCLUSÕES:**

Avaliações de programas e serviços realizadas no âmbito da Comissão Intergestores Regional, partindo do interesse local e facilitando o envolvimento de seus membros pelo uso de metodologias participativas, podem contribuir para a construção da integralidade do cuidado. Na medida em que o ato de avaliar fique investido de maior significado para os atores locais, seu envolvimento com as avaliações em nível federal poderá ser também estimulado.

## INTRODUCTION

The evaluation in health remains quite studied and discussed in the world scenario^10^. The crisis in the health sector calls for reflection on the results achieved to improve the performance of the system^5^, and the demand for transparency in the management of public resources remains a priority on the agenda.

In Brazil, the publications approach many aspects of this topic with considerable depth. There are studies on the constitution of the field of evaluation^14^, on the process of institutionalization of the ongoing evaluation in the Country^8^, and reports and robust analyses of experiences carried out^3,12^.

Within the Brazilian Ministry of Health (MS), a very fruitful period for the field took place between 2003 and 2008, represented by the Programa de Expansão e Consolidação da Saúde da Família (PROESF – Program of Expansion and Consolidation of Family Health). Together with the establishment of the National Policy for Evaluation of Primary Health-care, which sought to solidify the process of institutionalization of the evaluation under the perspective of decentralization, a great investment was carried out, which, among other actions, has enhanced the creation of collaborating centers in evaluation in different institutions of higher education in the Country and encouraged a large reflective process on the topic^9,15^.

Currently, the Department of Monitoring and Evaluation of SUS (DEMAS)^a^, created in 2011, set the establishment of a “Evaluation System for the Qualification of SUS”^20^, composed of evaluation instruments such as the *Programa Nacional de Melhoria do Acesso e da Qualidade da Atenção Básica* (PMAQ – National Program for Improving Access and Quality of Primary Health-care)^b^ and the *Índice de Desempenho do Sistema Único de Saúde* (IDSUS – Index of Performance of the Unified Health System)^c^, with the aim of “producing, by evaluations, a set of necessary and strategic information to the development and qualification of SUS”^22^.

We placed such initiatives as part of “monitoring and evaluation macrosystems”^16^, aimed at producing information of broad thematic scope, with enough depth to the management monitoring of the programs, allowing us to analyze their performance and set the necessary adjustments to the policies. Aimed at health-care at the federal level, they offer the same evaluation format for all regions of Brazil, using the same judging criteria, thus complicating the decentralized decision-making and, therefore, the adjustment of the programs to the different contexts in which they develop.

We can observe, in the field, a gap in the study of evaluations carried out at the local level, aimed at achieving a deeper level of understanding of the organization of services, including relationships between certain contexts and formats.

The Brazilian Unified Health System (SUS) idealizes the strengthening of the local management^24^, and processes to stimulate the development of territorial governance are in progress. The study of local evaluations would add tools in this same direction.

It is important, moreover, to point out the special difficulty in finding studies on methodologies and structuring of evaluations that move away from the standardized application of normative and quantitative indicators and that take as a basis different labels for different sociocultural contexts^2^.

The evaluation, especially of participatory approach, can become an instrument of learning for local actors, to the extent that it addresses their questions and contribute with relevant information to decision-making, allowing to expand the potential for changes in municipal practices of work and management.

Many definitions of “participation” and classifications of “participatory approaches” exist. However, one aspect that seems common to all of them, and is one of the features of the process that will be analyzed in this article, is the emphasis on the horizontal partnership established between evaluation specialists and people interested on the object evaluated, aiming at the joint production of evaluative knowledge.

Since we worked with a group of managers, we decided to use the Pragmatic Participatory Evaluation referential^6^, centered on facilitating the use of the evaluation process in the instrumental (support to decision-making), conceptual (focused on the actors’ learning), and political dimensions (facilitating negotiations between partners).

Routinely, the attention of municipal health managers is monopolized by emergency situations in the technical and political context, as well as by information demands of the state and federal levels, rather than by reflexive actions of planning and evaluation. Often, the lack of professional training, especially in small cities^21,25^, is also associated with the restricted use of these two management tools by them.

This study analyzed the application of a methodology for evaluation at the local level and in participatory basis, focused on understanding the meaning that the organization of health services acquires for the regional actors. We assume that the complementarity of this model with the initiatives at the federal level will allow a potentiation of both.

## METHODS

We used the methodology of single case study^26^, which allows enhancing the discussion on evaluation strategies at the local level, as well as identifying significant topics for the organization of health services that are common to the diversity of existing contexts.

The studied health region belongs to the state of Sao Paulo and was chosen because of potentially favorable factors for the feasibility of the research and that are present in several real spaces of SUS implementation: (i) policymakers and managers with an active participation in the Regional Interagency Commission (CIR) and in process of group strengthening, potentially increasing their sensitivity as to the utility of evaluative processes; (ii) reasonable installed capacity in the health area – taking all the cities –, constituting a variety of elements that would tend to enrich the process of formulation of evaluative questions.

A group with managers who participate of CIR was formed, from seven^d^ of the 18 cities that constitute it. The proposed goal was to evaluate a specific problem in a line of care provided by the health region, which would be chosen by them later.

The proposal was presented in one of the meetings of this body and the accession of managers was voluntary. There was a direct invitation to specific cities, made by participants that were first interested by the proposal, to form a diverse group in terms of population size and presence or absence of carceral and indigenous population.

As already mentioned, the process was developed based on a participative methodology, understood here according to the Cousins and Whitmore referential^6^. This choice led all the decisions of the process and aimed to ensure the relevance of the process to the participants, creating a space for learning and facilitating the use of evaluative findings.

For triggering activities, some activities with defined goals have been proposed to the group (Table 1). From this, the steps that followed were based on the participants’ demands.

**Table 1:** Initial proposals and justifications of the evaluation process implemented in the health region. Sao Paulo, Southeastern Brazil, 2014.

Proposal	Justification
Approval of the evaluation project by CIR	Value CIR as main forum of discussion of issues relating to the health region. Enhance the perspective of institutionalization of the evaluation process
Initial production, by all the municipal representatives of CIR, of a listing of “discomforts”* of everyday decision-making	Enable the inclusion of CIR members not directly participating in the process. Facilitate the subsequent use of the findings of the evaluation by a larger number of policymakers and managers
Choice of the theme of the evaluation in a connected way with the agenda and with the efforts already underway in the cities, as well as with the needs of the health region	Legitimize the choice before CIR

CIR: Regional Interagency Commission

* Aspects considered by them as able to be better dealt with if they could dispose of diverse information or tools.

The topic initially chosen by the group for the evaluation was the flow of users between the primary health-care and the medium and high complexity health-care. Because of the coverage and diversity of the specialties involved, the elective general surgery has been identified as tracer of the process (according to Kessner’s et al.^17^ definition). Then, the group focused in cholecystectomy surgeries, according to the greater number of cases and long waiting time, aiming at a non-exhaustive data collection, but that would bring information about the main aspects to be evaluated and with potential in the decision-making of local managers.

Seven workshops were held and the steps of construction of the evaluation listed in Table 2 were covered. All workshops were recorded and the audios, transcribed.

**Table 2:** Construction of the evaluative process: actions, motivators, and topics discussed in the workshops carried out in the health region. Sao Paulo, Southeastern Brazil, 2014.

Action	Motivators	Main topic discussed	Workshops in which they have developed
Initial choice of the topic of the evaluation	Knowledge of the interests of participants, feasibility, and usefulness of the evaluation	Integrality of care	1
Data collection on the offer of specialties in the the CROSS System	Presence of a concrete object of analysis Preview of the process requirements	Use of the offer of specialties. Negotiation with AME (access to the waiting list, referral mechanisms)	1
Request of AME users’ listing waiting for surgery by the state hospital	Information unavailable and considered essential by municipal managers to further understanding the situation to be modified	Need for information to enable flow management	Between workshops 2 and 3
Definition of the evaluative questions and indicators	Visualization of the evaluation process as a whole Guarantee of linkage to issues that have meaning for the group	Value judgment	1,2,3
Joint access and study of data stored in SIH	Discussion of parameters for judging the adequacy of the offer in each of the levels of complexity	Flow for specialties. Topicality of Ordinance 1,101^a^	3,4,5
Primary qualitative data collection (7 focus groups and 8 in-depth interviews with users from five of the cities participating In total, 28 users were listened to^b^)	Selection of topics directed at specific contents aiming at the close connection between the topic of the group and the object of the evaluation, to preserve the legitimacy of the approach with the user	Stage started after the initial mapping of the problem: the dialogue with the user brings in itself the commitment of forwarding the demand identified by the study results	3,4,5
Interview with the director of AME	Comprehension of access and the flow of this secondary level of health-care in the territory	Negotiations between government levels and private providers	Between workshops 5 and 6
Report and discussion of the primary data collected	Knowledge of the user’s perspective	User satisfaction with primary health-care User told about the urgency of his medical condition (gallstones) but not about the waiting time for the surgery	6
Communication of results	Analysis of all data collected	Possibilities for action	7

CROSS: *Central de Regulação de Oferta de Serviços de Saúde* (Coordination Center of Offer of Health Services); AME: *Ambulatório Médico de Especialidades* (Outpatient Department of Specialties); SIH: *Sistema de Informações Hospitalares* (Hospital Information System)

^a^ Brazilian Ministry of Health. Ordinance no. 1,101/GM, June 12, 2002. Establishes parameters of assistance coverage within the Brazilian Unified Health System – SUS [Internet]. Brasilia (DF); 2002 [cited 2015 Jun 8]. Available from: http://www1.saude.ba.gov.br/regulasaude/2009/PN%20PORTARIAS%202009/nvos%20pdfs%202009/PT%20GM%201101%2012.06.2002.pdf

^b^ All users were waiting for vacancy of cholecystectomy or had performed the surgery at most two years before. The collection focused on the description of the path of these users, since the perception of the symptom until the resolution of the problem with the surgery.

The empirical material was analyzed by hermeneutic-dialectic methodology^19,20^. This approach preserves the richness of the collected qualitative material and values the context of the health region where the evaluation took place.

This study was approved by the Ethics Committee from the Faculdade de Saúde Pública of Universidade de São Paulo – FSP/USP (Opinion 1,006,380). All participants signed the informed consent form.

## RESULTS AND DISCUSSION

The use of pragmatic participatory methodology^6^ allowed the workshops to settle in a democratic way and to be directed by the direct interests of policymakers and managers. The gathered empirical material showed three results that portray the managers’ appropriation of the process and that are configured as axes of analysis:

- Deepening the understanding regarding the organization of adult health-care in the defined region;
- Appropriation of tools for survey and analysis of quantitative and qualitative data;
- Possibilities for action.

Below, the item related to the replication of the evaluation process is presented, which also emerged from the participants’ suggestions, and arises here as one of the main limitations to the use of the methodology.

### Deepening the Understanding Regarding the Organization of Adult Health-care in the Defined Region

Facilitating a highly reflective space for managers and keeping proximity to everyday problems, the process undertaken increased the participants’ view about the dynamics of SUS’s current operation, whose discussion usually do not have space in the predominantly administrative CIR meetings^21^.

The choice of elective surgeries portrayed the concern with the construction of integral care, directly linked to the possibility of intergovernmental negotiation. The group discussed the autonomy of municipal managers facing decisions and inductions of policies coming from the state or federal management^18^ and the division between the different realms of management and the different rationales present in the field – funding logic, understanding of the role of the federated entities in the organization of the system^11^. They also approached the practice of decentralization that occur in the Country without the necessary planning for prior expansion of the administrative and institutional capacity of the cities^23^.

Our survey has many punctual demands, such as “urgency and emergency” and “difficulty in the access to medium complexity”. If we have to start by topics that are under the governance of cities, I would say that none of them is. It’s no use just the cities. The state has to work for the whole system work... It’s a gear, a machine... (Municipal Manager 1)

We here, facing a hospital bed crisis... You should have a shared system, of partners that are involved in the same problem, and what’s the answer that we have from the state government? “It’s a ’municipal fault, which doesn’t invest in hospital care”. So, how can we deal with this? (Municipal Manager 2)

But I doubt if this is because we have no structure... Who conducts the surgeries? It’s mainly the State Hospital for city X and for the others. With what logic does the city works, and, to some extent, as consequence, the AME [Outpatient Department of Specialties] itself works? With the logic of the population needs. With what logic does the State Hospital works? With the logic of terms they made. They’re going to conduct 500 surgeries, “oh, but I have 3,000 patients”. Then he thinks: “If I do this here, we’re going to spend such amount of money”. They have an economic system there... (Municipal Manager 3)

At the time of analysis of the data collected among the users, they discussed the need for systematic collection and use of this type of information in the management. They emphasized that the acquaintance of the experiences lived by the main interested ones in the service^1^ makes the discussions more significant and allows the deepening of the analyses.

They unfolded the discussion to another facet of integral care: the consideration to the integral being of the user and to the health needs brought by him^4^, including the management of directions and the provision of information about the operation of the services.

The doctor, when we pass, says “there’s a bomb inside you”. And then you go to the line... Who sleeps like this? (User 1)

I had a crisis that I thought I was going to die, I was taking saline at the prompt service... That’s when I got in touch with the doctor of my city, because I knew he operated for another city, and I went after him. I paid the consultation to see him and he operated on me in the other city, and by the SUS! If I had waited for AME, I would be dead today. (User 2)

The user, in addition to being in the hands of two managers (state and municipal), one does not know what the other is doing. Neither the user or us know where he is on the waiting list, which is the prediction of his attendance. (Municipal Manager 1)

### Appropriation of Tools for Survey and Data Analysis

Managers and administrators often stated that the information available to the city regarding the user’s path always stopped when the patient was referred to the equipment under state management. An initial joint study of the reports provided by the CROSS system (Coordination Center of Offer of Health Services) showed the usefulness of an effort of appropriation of the information contained there, often devalued, or even unknown, by the managers. Similarly, the review of internal processes of the city also stood out. Information that could be collected in the municipal regulatory services were routinely lost.

Then she just didn’t go see a surgeon in our municipal network, but simply went to AME. Then I managed to get into the AME system. I can’t figure out why she stayed only in the AME. (Municipal Manager 2)

They used the Tabwin program, provided by the Ministry of Health to consult the data from the Hospital Information System, and the majority of managers used it for the first time. They valued the gains and argumentations with partners, which arised from the generated information.

The group observed that both the work done by AME and the reference State Hospital took place autonomously regarding the actions of primary health-care. Each federated entity preserves its autonomy, focusing on the role formally granted to it, and not on the networking.

To do this reasoning, one had to add the urgency that you make in the city to the urgency that the State makes. If the offer of the elective was higher, the urgency would be smaller. (Municipal Manager 2)

You will come to the conclusion that what is agreed within the management contract with the provider is not answering the need... If neither of our Collegiate Body, let alone the whole network in which the state is a reference... (Municipal Manager 1)

### Possibilities for action

In the workshops, the group noted that any proposal for a change in the system would involve a review of all services available under the different levels of management in the territory – including their own. The review of the primary and emergency units while referral sources, of the regulation and use of the CROSS system, as well as of the management of charitable hospitals, proved to be fruitful.

In the CROSS system, 48 hours before we can no longer change the name of the patient. If he gives up because of a misadventure, if a urgent last minute case appears, we cannot insert anymore... With that, he ends up missing the consultation. It was time to review that. (Municipal Manager 1)

Manager 4: In my city there is a Charity Hospital, but it doesn’t have the service... it’s basically a matter of providing. When we had a team of participatory providers, with credibility, which collected funds, it was one reality. After that, nobody else campaigned and basically the city hall maintains the Charity Hospital, then there’s an office that chooses someone and put him there, it’s all political office, nobody understands anything and it stays as it is.

Manager 2: You have to press the provider, as I am doing there, to hire technicians, people hired for career here and teach everything to continue, because otherwise every time a new administrator enters, we’ll have always to say the same things we say.

From reports of moments of political negotiation with partners, we realized that the group started to rely more on technical arguments and in-depth reflections, reflecting a maturing to make possible new action strategies. These are only initial strategies, but that reflect the expansion of proactivity to the establishment of a link between the different governmental actors and private partners.

CIR’s technical chamber has been strengthened as a space of exchange and negotiation with private providers, to the extent that it builds a more balanced power among the participants. Such balance results from the appropriation of knowledge of municipal managers about flows, degree of autonomy of each service, and mechanisms that are at stake in the agreement, among others. Obviously, important limitations remained, e.g., regarding decisions about the organization and terms of services of medium and high complexity health-care that occur centrally in the Health Secretariat of the State of Sao Paulo, which virtually precludes the involvement of municipal managers.

A possible repositioning of the role of existing hospitals in the cities, which would assume part of the demand for general surgery, was considered for new designs of regionalization of the care.

When we talked in the monitoring group of providers about increasing the number of elective surgeries, the hospital director said “we would need to study it, we don’t have more capacity... unless we open the third shift of surgery”, and then suddenly we enter in these possibilities, if we start pushing it somewhat... (Municipal Manager 1)

With the COAP [Organizational Contract of Public Action], we can covenant that it’s going to be the municipal hospital that will give vent to the region, that will attend, make this type of procedure... a negotiation between entities, both the municipal ones... and see which of them can give support. (Municipal Manager 1)

### Replication of the Evaluative Process

The respect to the pace of the group did not prevent that all structuring steps of an evaluation were completed in the process, generating appropriation of the “evaluative reasoning”. It is known that the very questioning of the “why”, “when” and “how” evaluate allows a critical analysis of the evaluated object in its complexity.

However, as expected, given the novelty of the process, the participants did not really believe that the process could be replicated regardless of the presence of the facilitators and of the contract that was made. They reiterated that the daily management is taken over by emergency issues and that there would be no room for the construction of this type of process, if it was not for the commitment made with the academy.

I think that before the diversity of the work that we have, I would hardly stop to analyze all the items, I don’t have time for that. I stopped because it was a directed work, of making a commitment... I’m saying that this thinking is not part of the routine. (Municipal Manager 5)

## CONCLUSIONS

It is essential to contextualize this study as the use of a methodology that stimulated the involvement of administrators and local managers in the search for answers to questions of direct interest. It was possible to start a process of recognition of the necessity, approximation and appropriation of information, supported by simple measures of data collection, analysis and reflection, which tend to strengthen the fragile situation of the evaluation in the decision-making process.

The participatory process brought consistency to the identification of variables that influence the service evaluated. The adjustment of the offer was identified as an alternative to improve the flow of assistance between primary health-care and medium and high complexity health-care, but the notion that this is not the only possible action was presented. As a result, new fronts of communication could open. The proactivity of policymakers and managers has increased in the search for negotiation with the other levels of management. Even during the process, minor changes, tending to facilitate the flow management, could be combined in person, in local meetings. Obviously, such changes have taken a local range: actors of different levels of management that establish more proximity and leverage changes.

We stress the procedural aspect of this initiative, which, as in any learning process, does not predict or require the reach of large and visible results in a short time.

We aim to contribute to the construction of a culture of evaluation in the local levels of the system, strengthening a proactive attitude of policymakers and managers in the search to use the information for the enrichment of the political processes. Even if supported by the constant presence of the academy, at least in its early stages, the proposed evaluation model enhances evaluations carried out on the macrosystemic level. To discover, for the evaluation, a meaning more connected to improvements in the concrete practice, making it less prescriptive and bureaucratic, can facilitate its transposition into the comprehension of broader spheres.

Important limitations arise from the lack of institutionalization of the process, which had limited organizational insertion. However, experience shows that we hardly begin from ideal conditions: they are built from bottom-up processes.

In that sense, in democratic processes, the matter of participation is exemplary regarding the division between theory and practice. There is a consensus in methodologies that advocate the involvement of different interested entities in the evaluation: that the multiplicity of points of view enriches the construction and analysis of the evaluated process, and tends to leverage the use of its results^7,13^. The process in focus has allowed us to identify that the conditions for such participation to occur are not yet in the public service. It takes effort to enhance its construction, in the sense of negotiation in action priorities, building of credibility, and gradual maturation of the process.

## Footnotes

a Ministério da Saúde, Secretaria-Executiva, Subsecretaria de Assuntos Administrativos, Coordenação-Geral de Gestão de Pessoas. Por dentro do Ministério da Saúde: orientações aos novos servidores. Brasília (DF); 2013. Available from: http://bvsms.saude.gov.br/bvs/publicacoes/por_dentro_ministerio_saude_orientacoes.pdf

b Ministério da Saúde, Secretaria de Atenção à Saúde, Departamento de Atenção Básica. Programa Nacional de Melhoria do Acesso e da Qualidade da Atenção Básica (PMAQ): manual instrutivo. Brasília (DF); 2012. (Série A. Normas e Manuais Técnicos). Available from: http://189.28.128.100/dab/docs/publicacoes/geral/manual_instrutivo_pmaq_site.pdf

c Ministério da Saúde. Índice de Desempenho do SUS – IDSUS. Brasília (DF). Available from: http://idsus.saude.gov.br

d Participated in the research: the hub city of the region, class VI of population size (from 100,001 to 500,000 inhabitants), a city of class V (from 50,001 to 100,000 inhabitants), two cities of class IV (20,001 to 50,000 inhabitants), two cities of class III (10,001 to 20,000 inhabitants) and one city of class I (up to 5,000 inhabitants), according to the classification used by IBGE/2011.
